# Utilising bioinformatics and molecular docking technology to explore the underlying mechanisms of intervertebral disc degeneration with potential therapeutic drugs and formulas

**DOI:** 10.7189/jogh.15.04298

**Published:** 2025-11-21

**Authors:** Tian Yukui, Cui Xiaofeng, Bai Xue, Guo Lei, Wang Cheng, Liu Junchang

**Affiliations:** 1 College of Traditional Chinese Medicine, Xinjiang Medical University, Urumqi City, China; 2 Massage Department, Xinjiang Uyghur Autonomous Region Institute of Traditional Chinese Medicine, Urumqi City, China; 3 The Fourth Clinical Medical College, Xinjiang Medical University, Urumqi City, China

## Abstract

**Background:**

Intervertebral disc degeneration (IDD) is prevalent in orthopaedics, yet lacks effective treatments. This study seeks to discover potential therapeutic targets for IDD to inform clinical therapies and traditional medicine approaches.

**Methods:**

In this study, IDD-related data sets were sourced from the Gene Expression Omnibus, and differential expression analysis was performed to identify differentially expressed genes. Subsequently, candidate genes associated with IDD were recognised using databases such as GeneCards, OMIM, DrugBank, and DisGeNET, with further validation of these genes' biological functions and involvement in signalling pathways through enrichment analyses. Additionally, machine learning algorithms were applied to select candidate targets. The diagnostic value of these targets for IDD was assessed by constructing a nomogram model, and their functional networks and biological processes were revealed using GeneMANIA and Gene Set Enrichment Analysis. Eventually, the research also encompassed immune infiltration analysis and the construction of competing endogenous RNA (ceRNA) networks, as well as predictions for potential drugs and traditional Chinese medicine (TCM) prescriptions.

**Results:**

A total of 89 differentially expressed genes were identified through bioinformatics analysis, and further analysis led to the determination of 16 candidate genes associated with IDD. Seven candidate targets were found from the candidate genes using machine learning methods. Two of these targets, cytochrome P450 family 1 subfamily B member 1 (CYP1B1) and tumour necrosis factor alpha-induced protein 6 (TNFAIP6), were chosen as key targets because they demonstrated a significant difference in expression in IDD. Following, it was also found that CYP1B1 and TNFAIP6, as well as the nomogram, indicated good predictive performance for IDD. Furthermore, gamma-delta T cells were more prevalent in IDD. CYP1B1 and TNFAIP6 showed strong correlations with gamma delta T cells, indicating a tight link between these key targets and the pathology of IDD. Eventually, 11 natural small molecules corresponding to key targets were discovered. Three of these compounds (Quercetin, Genistein, Apigenin) were found in six TCM. This could offer new theoretical references for the clinical treatment of IDD.

**Conclusions:**

This study identified CYP1B1 and TNFAIP6 as important targets for IDD, developed a predictive nomogram, and explored the application of TCM herbal formulae, providing new insights into the clinical treatment and prescription development of IDD.

Intervertebral disc degeneration (IDD) has become increasingly common in aging societies, seriously affecting patients' quality of life. For countries experiencing pronounced population aging, such as China, IDD imposes substantial medical costs and productivity losses, with an increasingly younger onset age [1–3]. The intervertebral disc (IVD) is a non-vascular structure in the adult body that consists of the nucleus pulposus (NP), peripheral anulus fibrosus, and upper and lower discs [4,5]. Degenerative changes often include decreased water in the NP of the lumbar disc and degeneration of the NP, leading to lumbar disc herniation or other complications [6]. From a pathological perspective, IDD develops due to a complex interplay of factors such as aging, oxidative stress, inflammation, and mechanical stress [7,8].

Currently, IDD is treated both conservatively and surgically, depending on the patient's symptoms. However, these methods only relieve pain but do not reverse IDD and rebuild the mechanical function of the spine. Recent research is moving towards the field of biological therapies such as mesenchymal stem cells (MSCs) and non-coding RNA [9]. However, its clinical translation still needs to overcome many biological and technical difficulties to ensure its safety and efficacy in clinical applications [9]. Because the pathogenesis of IDD remains unclear – particularly the lack of systematic elucidation of key driver genes and signalling pathways – developing targeted therapies has proven challenging [10]. Current treatments, including conservative management and surgical interventions, can only alleviate symptoms but cannot reverse the pathological process or restore spinal function [11]. Although biological therapies, such as MSCs or non-coding RNAs, hold promise, they face significant barriers to clinical translation, including safety validation, delivery efficiency, and uncertainty regarding long-term efficacy [12]. Traditional Chinese medicine (TCM) emphasises the integration of the interrelationship between diseases and drugs, which is not only based on rich practical experience, but also has the advantages of fewer adverse reactions and milder drug effects [13]. The unique diagnostic and therapeutic approach of TCM, particularly its synergistic multi-component, multi-target, multi-pathway method, holds significant clinical value in alleviating symptoms of low back pain and mobility issues while slowing disease progression. Additionally, TCM is recognised for its favourable prognosis and high clinical safety, making it a current focus of medical research [14].

Traditional Chinese medicine (TCM) has been utilised to treat numerous conditions, including IDD, for thousands of years across China, Japan, South Korea, and other Asian nations. Analysis of formula composition reveals that Danggui, Chuanxiong, Duzhong, Dihuang, Duhuo, Xuduan, and Sangjisheng are the most frequently used herbs. The therapeutic mechanisms of TCM formulas for IDD primarily involve reducing the release of inflammatory mediators, decreasing oxidative stress, and preventing apoptosis in nucleus pulposus cells (NPCs) by modulating the expression of specific genes or proteins within relevant pathways [13]. Previous studies have demonstrated that components of TCM act synergistically to regulate IDD. Active compounds in TCM formulae, such as Bushen Huoxue Decoction, including quercetin, genistein, and apigenin, have been shown to modulate key IDD-related pathways via multiple targets. For instance, quercetin, abundant in *Eucommia ulmoides* and *Taxillus chinensis*, suppresses oxidative stress and inflammatory responses by activating the nuclear factor erythroid 2–related factor 2 (Nrf2)/nuclear factor kappa-light-chain-enhancer of activated B cells (NF-κB) signalling axis [15]. Genistein, enriched in Carthamus tinctorius and Pueraria lobata, mitigates apoptosis by inhibiting p38 mitogen-activated protein kinase (p38 MAPK) phosphorylation [16]. Apigenin, present in *Bupleurum chinense* and *Scutellaria baicalensis*, restores autophagic flux through the AMP-activated protein kinase (AMPK)/mechanistic target of rapamycin (mTOR) pathway [17]. These natural compounds not only target individual IDD-related pathways, such as chemokine signalling and focal adhesion (FA), but also modulate the immune microenvironment – such as gamma delta T cells (γδ T cell) infiltration – to slow the degenerative process [18].

Current research on TCM for the treatment and intervention of IDD has predominantly focused on pharmacological mechanisms, with observational and preclinical studies showing significant symptom relief [19]. Traditional Chinese medicine (TCM) therapy for IDD primarily targets pain and joint mobility impairment, achieving effects such as promoting blood circulation, removing stasis, and relaxing tendons through the oral administration of herbal formulae [13]. However, the molecular mechanisms underlying IDD have not been fully elucidated and remain in the preliminary exploratory stage. Most studies concentrate on pharmacological phenomena, lacking in-depth validation of interactions between active components and their molecular targets [19]. Moreover, TCM research faces certain limitations, including variability in herbal composition, incomplete quality standards, insufficient high-quality large-sample randomised controlled trials, and the complexity of formulae leading to unclear therapeutic targets [13,20,21]. Despite these challenges, TCM retains unique value in the management of IDD. Therefore, elucidating the molecular mechanisms of individual herbs and formulae in IDD treatment remains of considerable practical significance.

In this study, we applied bioinformatics approaches, integrating multi-omics analyses of IDD transcriptome data to identify key targets and construct a nomogram model for predicting IDD risk. Through network pharmacology, we analysed protein-protein interaction (PPI) networks and immune infiltration associated with these key targets to investigate the molecular mechanisms of IDD further. Finally, molecular docking was employed to verify the binding activity between natural compounds and the identified targets, predicting potential drugs and TCM formulae relevant to IDD treatment. This work provides a novel theoretical framework for integrated ‘gene-compound-formula’ therapy for IDD. In the subsequent research, we will perform in vitro and in vivo molecular mechanism studies on the identified key targets, followed by animal model validation and, ultimately, clinical trials to evaluate their therapeutic potential and safety.

## METHODS

### Collection of data

Gene expression profile data sets GSE70362 (platform GPL17810) and GSE56081 (platform GPL15314) were retrieved from the Gene Expression Omnibus database [22]. The training set (GSE70362) comprised 10 disc nucleus samples from IDD patients and 14 from normal controls, while the validation set (GSE56081) included five disc nucleus samples from IDD patients and five from normal controls. After downloading the raw data, sequencing quality was assessed using FastQC, v0.11.9 (Babraham Bioinformatics, Cambridge, UK), and samples with an RNA integrity number <7 were excluded [23] to ensure that transcript integrity met the analytical requirements. This publicly available data set is tissue-specific, providing primary human NP samples and thereby avoiding interference from gene expression of other spinal structures, such as end plates or ligaments. The data set also contains complete phenotypic information, including well-defined Pfirrmann grading for group classification (normal *vs*. IDD), facilitating differential expression analysis. A schematic diagram illustrating the analysis workflow is provided (Figure S1 in the **Online Supplementary Document**).

### Differential expression analysis

The limma package (v 3.50.1) [24] was used to identify differently expressed genes (DEGs) between IDD and control samples in GSE70362 (|Log_2_ fold change (|Log_2_FC||>1, *P* < 0.05). The volcano map and the heat map were constructed by the ggplot2 package (3.3.5) [25] and the ComplexHeatmap package (v 2.10.0) [26], respectively.

### Identification and function analysis of candidate genes

To identify additional disease targets linked to IDD, searches were conducted in the GeneCards database (https://www.genecards.org/), Online Mendelian Inheritance in Man (OMIM) database (https://www.omim.org/), DrugBank database (https://www.drugbank.ca/), and DisGeNET database (https://www.disgenet.org/) for IDD-associated targets. The gene data retrieved from the four databases were combined, resulting in a set of IDD-related disease targets. The DEGs and IDD-related disease targets were intersected using VennDiagram, with the overlapping portion defined as candidate genes. Subsequently, the biological functions of candidate genes were examined through Gene Ontology (GO) and Kyoto Encyclopedia of Genes and Genomes (KEGG) enrichment analyses using the clusterProfiler package (v4.6.0) [27], with a significance level set at *P* < 0.05. Moreover, a PPI network (interaction scores >0.15) was constructed using the STRING database (http://string.embl.de/) to investigate interactions among candidate genes at the protein level.

### Identification of key targets

To further screen key targets, the Boruta package [28] was used to perform Boruta analysis on the candidate genes. To enhance the robustness of target selection, the mlbench and caret packages (v6.0-91) [29] were jointly applied to implement Support Vector Machine-Recursive Feature Elimination (SVM-RFE) for feature selection among the candidate genes. The VennDiagram package (v1.7.1) [30] was then used to obtain the intersection of the gene sets generated by the two methods. Boruta excels at comprehensively identifying relevant features, whereas SVM-RFE ranks features according to their classification contribution. Their intersection balances selection breadth and precision; thus, we defined the intersecting genes as high-confidence candidate targets. During the SVM-RFE process, 10-fold cross-validation was performed to reduce overfitting risk. In each iteration, the training set was randomly divided into 10 equal parts, with nine parts used for training and one part for validation, repeated 10 times, and the mean accuracy was calculated. Model performance was monitored using classification accuracy and the area under the curve (AUC). Iterations were terminated when further feature reduction resulted in a significant decline in performance. If discrepancies arose between the two methods, only the intersecting genes were retained as high-confidence candidate targets. Non-overlapping genes were subjected to expression trend validation (IDD group *vs*. control group), and only those showing significant differences in both the training and validation sets (*P* < 0.05) were preserved.

To understand disease-related expression trends, candidate targets' levels in IDD *vs*. control samples were tested (*P* < 0.05), and significantly differing candidates with consistent trends across GSE70362 and GSE56081 were termed key targets, with box plots drawn for analysis. Subsequently, to ascertain the diagnostic value of key targets for IDD, the presence or absence of IDD samples in GSE70362 and GSE56081 was utilised as the outcome variable. The receiver operating characteristic (ROC) curves for individual key targets were plotted using the pROC package. At once, for the prediction of the risk of IDD occurrence based on key targets, nomograms were constructed using the rms package, with IDD samples being considered as the outcome event. In addition, calibration plots, ROC curves, and decision curve analysis associated with the nomograms were generated to evaluate the model's performance.

### Functional analysis of key targets

Other genes related to the functionality of key targets were predicted using GeneMANIA (http://www.genemania.org/), and the functions in which these genes participate were also identified. To elucidate the roles of key targets in disease progression, Spearman correlation coefficients were computed for key targets against all genes in IDD from GSE70362, then ranked to create gene lists. Subsequently, based on the ‘c2.cp.kegg.v7.4.symbols.gmt’ gene set, the clusterProfiler package (v4.6.0) [27] was used to perform Gene Set Enrichment Analysis for each key target. The false discovery rate method was applied for *P*-value adjustment, with statistical significance defined as an adjusted *P*-value <0.05 and an absolute normalised enrichment score (|NES|)>1. Finally, the enrichplot package (v 1.14.2) [31] was utilised to visualise the top five signalling pathways from the enrichment results of the key targets, which were sorted according to their enrichment scores.

### Immune infiltration analysis

To examine immune cell infiltration differences between IDD and control groups, the CIBERSORT algorithm (v 1.03) [32] assessed 22 immune cell types in both groups' samples from GSE70362. The Wilcoxon rank-sum test compared infiltration abundance, with *P* < 0.05 indicating significant differences. Following this, correlations between immune cells and key targets were analysed, calculating coefficients and significance, followed by plotting the correlation.

### Construction of network

The microRNAs (miRNAs) for the key targets were predicted utilising the miRDB (http://mirdb.org/) and TargetScan databases (http://www.targetscan.org/). Subsequently, the long non-coding RNAs (lncRNAs) associated with these miRNAs were predicted through the miRNet (http://www.mirnet.ca/) and StarBase databases (http://starbase.sysu.edu.cn/), and the intersection of lncRNAs from these databases was determined. This process culminated in the derivation of a network detailing the interactions between key targets, miRNA, and lncRNA. The competing endogenous RNA (ceRNA) regulatory network was then depicted using Cytoscape (v 3.7.0) [33].

### Prediction of key target-related drugs and herbal prescriptions

To bridge gaps in IDD treatment using TCM, potential drugs were predicted through DGIdb (https://dgidb.genome.wustl.edu/), and the Comparative Toxicogenomics Database (https://ctdbase.org/) was used to identify relevant small-molecule compounds for key targets. The SymMap database (http://www.symmap.org/) was utilised to find associated natural molecules and TCM symptoms with IDD, with results intersected with Comparative Toxicogenomics Database to identify potential compounds. The Traditional Chinese Medicine Systems Pharmacology Database and Analysis Platform database (http://tcmspw.com/) then helped ascertain TCMs, containing these compounds. Classic prescriptions from a list by the China Food and Drug Administration, containing over three of these TCMs, were considered candidate prescriptions. Cytoscape (v 3.7.0) [33] was used to visualise the network of potential compounds, TCMs, meridian tropism, and candidate prescriptions.

### Molecular docking analysis

To analyse the feasibility of treating IDD through key targets, the key targets and their corresponding potential compounds were subjected to molecular docking analysis. The crystal structures of proteins for each key target were obtained from the Protein Data Bank (https://www.rcsb.org/), and the 3D structures of active compounds were sourced from the PubChem database (https://pubchem.ncbi.nlm.nih.gov/). The activity of protein-compound binding was predicted using CB-Dock (http://cao.labshare.cn/cb-dock/), and molecular docking and visualisation of the key targets and active ingredients were performed using pyMOL.

### Data analysis

All analyses were conducted in the *R,* Studio v4.2.3 (Posit Software, Boston, Massachusetts, USA). All *R* scripts, custom functions, and parameter settings used in this study will be publicly available on GitHub upon publication. Intergroup differences were assessed using the Wilcoxon test.

## RESULTS

### A total of 16 candidate genes were obtained

In data set GSE70362 (IDD *vs*. control), 89 DEGs were identified, including 46 that were up-regulated and 43 that were down-regulated (Figure S2, Panels A–B in the **Online Supplementary Document**). Genes from the GeneCards, OMIM, DrugBank, and DisGeNET databases were compiled, resulting in 1675 IDD-related targets. The overlap between DEGs and IDD-related targets yielded 16 candidate genes (Figure S2, Panel C in the **Online Supplementary Document**).

According to the results of enrichment analysis, it was found that the candidate genes were mainly associated with GO entries such as cartilage development, extracellular matrix organisation, collagen-containing extracellular matrix, and extracellular matrix structural constituent, etc. (Figure S2, Panel D in the **Online Supplementary Document**). They were also enriched in KEGG pathways such as FA, extracellular matrix (ECM)-receptor interaction, Folate biosynthesis and Bladder cancer, *etc*. (Figure S2, Panel E in the **Online Supplementary Document**). Followed by, a PPI network was constructed, with an outlier gene being excluded, resulting in the identification of 15 genes with interactive relationships, which collectively encompassed 15 nodes and 49 edges. These relationships included the following pairs: IBSP (integrin-binding sialoprotein)-CNMD (chondromodulin), KRT19 (keratin 19)-CCND1 (cyclin D1), *etc*. (Figure S2, Panel F in the **Online Supplementary Document**).

### The key targets (CYP1B1 and TNFAIP6) were identified

By the machine learning algorithm, the final eight Boruta targets were identified, and they were CCND1 (cyclin D1), HYAL1 (hyaluronidase 1), TNFAIP6 (tumour necrosis factor alpha-induced protein 6), CYP1B1 (cytochrome P450 family 1 subfamily B member 1), IBSP (integrin-binding sialoprotein), PTHLH (parathyroid hormone-like hormone), COL6A2 (collagen type VI alpha 2 chain), and DPT (dermatopontin) (Figure S3, Panel A in the **Online Supplementary Document**). At once, a total of eight targets were identified using the SVM-RFE method, which were CCND1, CYP1B1, HYAL1, COL6A2, TNFAIP6, IBSP, PTHLH, and QDPR (Figure S3, Panel B in the **Online Supplementary Document**). After intersecting the two groups of targets, seven candidate targets were finally obtained, namely CCND1, CYP1B1, HYAL1, COL6A2, TNFAIP6, IBSP, and PTHLH (Figure S3, Panel C in the **Online Supplementary Document**). After validating the expression levels of the seven candidate targets in both the training and validation sets, two key targets showing significant and consistent expression differences were ultimately identified: CYP1B1 (training set: *P* = 0.012, Cohen's d = 0.84; validation set: *P* = 0.028, d = 0.81) and TNFAIP6 (training set: *P* = 0.006, d = 0.91; validation set: *P* = 0.017, d = 0.86) (Figure S3, Panels D–E in the **Online Supplementary Document**). These two genes were therefore defined as the key targets.

### The predictive effectiveness of CYP1B1 and TNFAIP6 was notable in IDD

In both the training and validation sets, the AUC values for CYP1B1 and TNFAIP6 were all greater than 0.7, indicating that CYP1B1 and TNFAIP6 had a high diagnostic value for IDD (Figure S4, Panels A–D in the **Online Supplementary Document**). A nomogram for predicting the risk of IDD based on the expression levels of CYP1B1 and TNFAIP6 was presented (Figure S4, Panel E in the **Online Supplementary Document**). The nomogram model depicted had good predictive value for IDD (Figure S4, Panel F in the **Online Supplementary Document**). Then, the AUC value of 0.922 for the nomogram, as revealed by its ROC curve, suggested that the nomogram model possessed good predictive value for IDD (Figure S4, Panel G in the **Online Supplementary Document**). However, this may have introduced a risk of model overfitting due to the limited sample size. In addition, it was also found that CYP1B1 and TNFAIP6, as well as the nomogram, had a certain distance from the two extreme curves, indicating high predictive performance (Figure S4, Panel H in the **Online Supplementary Document**). The C-index was 0.9219, suggesting extremely strong discriminative ability of the model, which could accurately predict patient outcomes. Moreover, the overall predictive performance of the nomogram was superior to that of a single variable.

### There was a high functional similarity between CYP1B1 and TNFAIP6

Based on the GeneMANIA database, 20 functionally related genes were predicted from two key targets (CYP1B1, TNFAIP6), namely EPHX2 (epoxide hydrolase 2), DCBLD1 (discoidin, CUB and LCCL domain containing 1), CYP1A1 (cytochrome P450 family 1 subfamily A member 1), GRM1 (glutamate metabotropic receptor 1), ESR1 (oestrogen receptor 1), ACAN (aggrecan), PLA2G4A (phospholipase A2 group IVA), CEBPB (CCAAT/enhancer-binding protein beta), NID2 (nidogen 2), HSPB2 (heat shock protein family B member 2), VCAN (versican), IL1B (interleukin 1 beta), SAE1 (SUMO activating enzyme subunit 1), FOS (Fos proto-oncogene, AP-1 transcription factor subunit), IL6 (interleukin 6), AHR (aryl hydrocarbon receptor), THBS1 (thrombospondin 1), CXCL8 (C-X-C motif chemokine ligand 8), FAP (fibroblast activation protein alpha), and CD44 (CD44 molecule, hyaluronan receptor). They exhibited various interactive relationships, encompassing processes such as eicosanoid metabolic process, extracellular matrix organisation, monocarboxylic acid biosynthetic process and fatty acid derivative metabolic process (Figure S5, Panel A in the **Online Supplementary Document**). Using Gene Set Enrichment Analysis, we identified the top five signalling pathways enriched in CYP1B1: chemokine signalling pathway, FA, neuroactive ligand-receptor interaction, cancer-related pathways, and actincytoskeleton regulation (Figure S5, Panel B in the **Online Supplementary Document****)**. While TNFAIP6 was primarily enriched in the following pathways: FA, huntingtons disease, leukocyte transendothelial migration, mapk signalling pathway and pathways in cancer (Figure S5, Panel C in the **Online Supplementary Document**). Previous studies have shown that the chemokine signalling pathway significantly enriched for CYP1B1 mediates the recruitment of macrophages to degenerated IVD via CCL2 (C-C motif chemokine ligand 2)/CCR2 (C-C motif chemokine receptor 2) and other molecules by regulating an upstream mechanism, thereby promoting a local inflammatory cascade [34]. In contrast, the FA pathway, predominantly driven by TNFAIP6, disrupts cellular mechanosensing through integrin-extracellular matrix (ECM) interactions, accelerating extracellular matrix degradation in NPCs [35]. These findings indicate that CYP1B1 and TNFAIP6 share substantial functional similarity in IDD diagnosis and represent key pathogenic target genes.

### Estimation of immune cell infiltrations in IDD

The infiltration levels of 22 types of immune cells were compared between IDD patients and normal controls (Figure S6, Panel A in the **Online Supplementary Document**). A Wilcoxon test revealed significant differences in two immune cell types (resting memory CD4+ (cluster of differentiation 4-positive) T cells and gamma-delta T cells) between the IDD and control groups (*P* < 0.05). Gamma delta T cells were more abundant in IDD samples, whereas resting memory CD4+ T cells were more prevalent in controls (Figure S6, Panel B in the **Online Supplementary Document**). In addition, CYP1B1 showed strong correlations with several specific immune cell types, including γδ T cells (Spearman's r = 0.58, *P* = 0.004), non-polarised macrophages (M0) (r = 0.47, *P* = 0.016), eosinophils (r = 0.43, *P* = 0.021), and memory B cells (r = 0.51, *P* = 0.009) (Figure S6, Panel C in the **Online Supplementary Document**). Similarly, TNFAIP6 was positively correlated with γδ T cells (r = 0.54, *P* = 0.007), M0 macrophages (r = 0.49, *P* = 0.012), and memory B cells (r = 0.52, *P* = 0.010) (Figure S6, Panel D in the **Online Supplementary Document**). The abnormal elevation of γδ T cells in IDD may exacerbate degeneration through two mechanisms:

(1) secretion of interferon-gamma (IFN-γ) to promote macrophage polarisation toward the pro-inflammatory M1 phenotype [36]

(2) activation of the NOD-like receptor family pyrin domain containing 3 (NLRP3) inflammasome to induce pyroptosis in NPCs [37].

The positive correlations between CYP1B1/TNFAIP6 and γδ T cells (Figure S6, Panels C and D in the **Online Supplementary Document**) suggest that these targets may form a ‘target-immune cell’ co-regulatory network that jointly drives the progression of IDD. These findings indicate a close association between the key targets and IDD.

### Regulatory network construction and potential drug prediction

Based on CYP1B1 and TNFAIP6, a total of 52 miRNAs were predicted. Subsequently, eight intersecting lncRNAs were obtained. Ultimately, an lncRNA-miRNA-mRNA network was established, which included one mRNA, four miRNAs, and eight lncRNAs. This network consisted of 13 nodes and 24 edges, with the following types of relationships: XIST (X-inactive specific transcript)-hsa-miR-543 (human microRNA-543)-TNFAIP6 (tumour necrosis factor alpha-induced protein 6), NEAT1 (nuclear enriched abundant transcript 1)-hsa-miR-142-5p (human microRNA-142-5p)-TNFAIP6 (tumour necrosis factor alpha-induced protein 6), *etc*. (Figure S7, Panel A in the **Online Supplementary Document**).

Besides, a total of 28 drugs with potential therapeutic value for IDD were predicted (**Table 1**). Then, 11 natural small molecules were identified from key targets, which include Resveratrol, Quercetin, Genistein, Progesterone, Apigenin, Emodin, Curcumin, Arsenic, and Monocrotaline. Specifically, the compounds Quercetin, Genistein, and Apigenin were associated with six TCMs: Eucommia Bark, Barbary Wolfberry Fruit, Safflower, Myrrh, Desertliving Cistanche and South Dodder Seed, used in the classic prescription 'Bushen Huoxue Tang'. Finally, a potential compound – potential TCM-meridian tropism – candidate prescription network was constructed (Figure S7, Panel B in the **Online Supplementary Document**). Through molecular docking analysis, it was found that the binding energies of CYP1B1 with Genistein, Apigenin, and Quercetin were −10.1 Kcal/mol (kilocalories per mole), −10.7 Kcal/mol, and −10.0 Kcal/mol (Figure S7, Panels C–E in the **Online Supplementary Document**), respectively. Meanwhile, the binding energy of TNFAIP6 with Genistein was −6.0 Kcal/mol (Figure S7, Panel F in the **Online Supplementary Document**). When the binding energy calculated by molecular docking is lower than −6 kcal/mol, the compound and protein are generally considered to have potential biological binding activity [38,39]. Therefore, our results indicated that the key target proteins CYP1B1 and TNFAIP6 exhibited strong docking interactions with their respective molecular compounds.

**Table 1:** Drugs with potential therapeutic value for IDD

Rank	Gene	Drug	Interaction score
1	CYP1B1	2'h,4h-(2,5′-bichromen)-4-one	4.212860993
2	CYP1B1	2'h,4h-(2,8'-bichromen)-4-one	4.212860993
3	CYP1B1	Chembl: chembl2347759	4.212860993
4	CYP1B1	Pinocembrin	2.106430496
5	CYP1B1	Zyc300	2.106430496
6	CYP1B1	Isopropyl alcohol	1.404286998
7	CYP1B1	Beta-naphthoflavone	1.404286998
8	CYP1B1	Formestane	1.053215248
9	CYP1B1	Fadrozole	0.842572199
10	CYP1B1	Aminoglutethimide	0.601837285
11	CYP1B1	Medroxyprogesterone	0.601837285
12	CYP1B1	Alpha-naphthoflavone	0.601837285
13	CYP1B1	Amodiaquine	0.526607624
14	CYP1B1	Toremifene	0.32406623
15	CYP1B1	Naringenin	0.300918642
16	CYP1B1	Anastrozole	0.300918642
17	CYP1B1	Chrysin	0.300918642
18	CYP1B1	Adjuvant	0.2808574
19	CYP1B1	Acacetin	0.2808574
20	CYP1B1	Exemestane	0.263303812
21	CYP1B1	Spironolactone	0.247815353
22	CYP1B1	Letrozole	0.221729526
23	CYP1B1	Epirubicin	0.203848113
24	CYP1B1	Rifampin	0.183167869
25	CYP1B1	Cyclophosphamide anhydrous	0.087767937
26	CYP1B1	Risperidone	0.041302559
27	CYP1B1	Fluorouracil	0.039743972
28	CYP1B1	Doxorubicin hydrochloride	0.01462799

## DISCUSSION

Intervertebral disc degeneration (IDD) forms the pathological foundation for spinal disorders and is a primary cause of low back pain. Research shows that approximately 60–80% of people worldwide experience low back pain, leading to substantial social and economic burdens [40]. Meanwhile, with the accelerating pace of population aging, IDD has become a global public health concern [41]. The IVD comprises the NP, peripheral anulus fibrosus, and end plate cartilage, which are vital for absorbing shocks, distributing and cushioning loads, and preserving spinal mobility. Intervertebral disc degeneration is the primary cause of disc herniation and related conditions, reflecting a gradual and progressive aging process [42]. Studies have shown that IDD is influenced and regulated by a variety of factors, such as mechanical load, autoimmunity, inflammatory factors and related pathways [43]. However, the systematic identification of core target genes driving IDD progression remains lacking [10], and significant challenges persist in the clinical translation of potential diagnostic biomarkers and therapeutic targets [19]. In this study, bioinformatics analysis identified two key targets: CYP1B1 and TNFAIP6. Both were found to cooperatively promote inflammatory responses and extracellular matrix degradation by regulating the chemokine signalling and FA pathways, respectively, and were positively correlated with elevated γδ T cell infiltration in the IDD immune microenvironment. In addition, both targets demonstrated high binding affinity with quercetin, genistein, and apigenin, three active compounds present in the ‘Bushen Huoxue Decoction.’ Our findings contribute to a deeper understanding of IDD pathogenesis and provide a theoretical basis for potential novel therapeutic approaches.

Traditional Chinese medicine (TCM) has a long history and is highly effective in treating complex and major diseases. Among these, TCM plays an essential role in the treatment of malaria and during COVID-19 [44-47]. Currently, opioids and blood-activating and analgesic medications are used for the treatment of IDD, which can alleviate some of the symptoms to a certain extent, but are prone to induce gastrointestinal adverse reactions, and to a certain extent, lead to poor patient compliance due to the long treatment period. At the same time, the prolonged treatment period leads to poor patient compliance to some extent, which in turn brings more difficulties to the treatment [48,49]. Traditional Chinese medicine has the advantages of multi-target and multi-pathway therapy, which can better achieve the purpose of treating IDD [50]. It shows good biological activity in inhibiting oxidative stress, anti-cellular aging, anti-inflammatory and analgesic, and regulating immunity [51,52].

This study established a ‘potential active compound-potential Chinese herb-candidate formula’ framework, further enriching the clinical diagnostic and TCM therapeutic system for lumbar disc degeneration. Based on multiple databases, DEGs were integrated with disease targets, and GO and KEGG enrichment analyses revealed several biological processes and signalling pathways associated with IDD. Using the Boruta algorithm in combination with SVM-RFE, seven candidate key targets were identified, among which CYP1B1 and TNFAIP6 exhibited the most significant expression differences and superior diagnostic performance. In addition, a nomogram constructed with these two targets underwent internal validation for CYP1B1 and TNFAIP6. The calibration curve demonstrated high concordance between predicted and observed risks, and the decision curve analysis confirmed substantial clinical net benefit. This model transformed gene expression levels into an intuitive IDD risk score, strengthening its potential for clinical translation.

Enrichment analysis indicated that the chemokine signalling pathway, strongly associated with CYP1B1, plays a critical role in IDD regulation. As a member of the cytochrome P450 family, CYP1B1 participates in oxidative stress by modulating reactive oxygen species metabolism [53], and its up-regulation exacerbates mitochondrial dysfunction in NPCs. Kyoto Encyclopaedia of Genes and Genomes (KEGG) enrichment also showed its involvement in cancer pathways, suggesting the accumulation of oxidative damage. CYP1B1-driven chemokine pathways, such as CCL2/CCR2, promote macrophage infiltration (Figure S5, Panel B in the **Online Supplementary Document**), consistent with the findings of Tian S, who reported significantly elevated levels of C-C motif chemokine ligand 2 (CCL2) and ligand 7 in myeloid cells under inflammatory conditions. Inhibiting CCL2/7 and its receptor, C-C chemokine receptor type 2 (CCR2), was shown to reduce macrophage infiltration and pro-inflammatory polarisation induced by myeloid cells [34]. In addition, several studies have similarly found the importance of chemokine pathways for IDD [34,54].

The FA signalling pathway, which is most closely associated with TNFAIP6, also exerts a significant impact on. IDD. Research has indicated that FAs are large molecular complexes made up of ECM, integrins, intracellular cytoskeleton, and various proteins. Acting as a mechanical bridge between the ECM and the cytoskeleton, these adhesions are essential for facilitating cell-environment interactions and regulating key processes by influencing diverse signalling pathways, both inward and outward, across different skeletal system cells [35]. The FA pathway mediated by TNFAIP6 (Figure S5, Panel C in the **Online Supplementary Document**) disrupts ECM-cytoskeleton force transmission via the integrin β1-integrin/focal adhesion kinase (β1-FAK) signalling axis, thereby accelerating the degradation of collagen II and proteoglycans [35]. As an inflammation-associated protein, TNFAIP6 directly responds to tumour necrosis factor-alpha (TNF-α) signalling [55] and, in IDD, this process potentiates the secretion of pro-inflammatory cytokines (*e.g*. interleukin-6 (IL-6) and interleukin-1 beta (IL-1β)) via activation of the nuclear factor kappa-light-chain-enhancer of activated B cells (NF-κB) pathway. Its role in orchestrating inflammation is further corroborated by KEGG enrichment analysis, which identified significant enrichment within the MAPK signalling pathway. Collectively, these pathways are implicated as key drivers in the pathogenesis of IDD, a conclusion strongly supported by our screening results.

Both CYP1B1 and TNFAIP6 have shown particular value in a variety of diseases. Weihang Li [56] highlighted that CYP1B1 is associated with lipid metabolism and that detection of CYP1B1 in the blood is potentially valuable for the early diagnosis of IDD. In addition, CYP1B1 also plays a role in endogenous metabolic pathways in metabolic disorders [53]. On the other hand, integration of myeloid proteomics and transcriptomics data identified TNFAIP6 as a possible candidate gene that significantly affects degeneration of IDD myeloid cells [57]. And TNFAIP6 was also observed to be up-regulated in the molecular response to IL-1β treatment in cells originating from NPCs, annular fibroblasts, and end plate cells [55]. Therefore, it is reasonable to suggest that the two key targets (CYP1B1, TNFAIP6) are involved in the core biological processes of IDD development.

This study found that CYP1B1 and TNFAIP6 are closely linked to specific immune cell types. CYP1B1 shows a significant correlation with gamma delta T cells, M0 macrophages, eosinophils, and memory B cells, while TNFAIP6 is significantly associated with gamma delta T cells, M0 macrophages, and memory B cells. The IVD acts as an immune-privileged organ, meaning the immune system identifies it as a foreign entity [58]. Immune infiltration, however, can significantly impact the progression of disc degeneration [36]. Immunoinfiltration analysis revealed that γδ T cells were significantly enriched in IDD samples (Figure S6, Panel B in the **Online Supplementary Document**) and positively correlated with the key targets CYP1B1 and TNFAIP6 (Figure S6, Panels C and D in the **Online Supplementary Document**). This finding has clear biological relevance: as innate immune cells, γδ T cells can promote macrophage polarisation toward the pro-inflammatory M1 phenotype in the degenerative disc microenvironment by secreting IFN-γ and TNF-α [36], and can also accelerate ECM degradation by inducing pyroptosis of NPCs through NLRP3 inflammasome activation [37]. Moreover, the strong correlation between M0 macrophages and the key targets (Figure S6, Panels C and D in the **Online Supplementary Document**) suggests a dynamic role in IDD. As non-polarised cells, M0 macrophages may differentiate into either M1 (pro-inflammatory) or M2 (repair) phenotypes. In a study investigating the role of myeloid cell-derived extracellular vesicles in macrophage function and metastatic regulation, up-regulation of CYP1B1 was found to suppress M2 macrophage phagocytic activity, which was considered critical for regulating M1/M2 macrophage function and promoting myeloid metastasis [59]. In the IDD inflammatory microenvironment, CYP1B1 and TNFAIP6 may drive polarisation toward the M1 phenotype via the CCL2/CCR2 axis (Figure S5, Panel B in the **Online Supplementary Document**), thereby enhancing the release of inflammatory mediators such as IL-1β and IL-6 and inhibiting cartilage matrix synthesis [34]. The increased infiltration of memory B cells (Figure S6, Panel C in the **Online Supplementary Document****)** is also noteworthy. Memory B cells may activate T cells by presenting self-antigens, such as ECM degradation products, thereby sustaining chronic inflammation [36]. Disruption of FA signalling mediated by TNFAIP6 (Figure S5, Panel C in the **Online Supplementary Document**) can release additional ECM fragments, such as fibronectin, triggering a vicious cycle of ‘ECM degradation → autoimmune response → inflammation amplification.’ Collectively, CYP1B1 and TNFAIP6 appear to drive the ‘inflammation-immune imbalance-ECM destruction’ axis in IDD by modulating γδ T cells, macrophage polarisation, and memory B cell activation. This discovery provides a new direction for IDD therapies targeting the immune microenvironment. Therefore, modulating immune cell activity through these key targets holds considerable therapeutic potential for IDD [37]. However, as a multifactorial degenerative disease, the pathogenesis of IDD extends beyond gene expression regulation and is also influenced by mechanical stress, environmental exposures, and the metabolic microenvironment. Because this study was based on static transcriptome analysis, it was unable to capture dynamic gene responses to mechanical stimuli – such as the mechanosensitive ion channel Piezo1 – or to reflect changes in metabolite concentrations and interactions between environmental factors and gene expression.

Subsequently, 11 natural small molecule compounds were obtained based on the key targets, among which three natural small molecule compounds (Quercetin, Genistein, Apigenin) corresponded to six TCM: Eucommia Bark, Barbary Wolfberry Fruit, Safflower, Myrrh, Desertliving Cistanche, and South Dodder Seed belong to the 100 Classic Formulas for Bushen Huoxue Formula (BSHXF). This formula has been shown to alleviate clinical symptoms significantly in patients with discogenic low back pain, including reductions in pain intensity and muscle tension, as well as improvements in function and lumbar spine mobility [60,61].

Studies have shown that BSHXF can lower inflammatory markers, including IL-6, IL-1, and TNF-α, in degenerative disc tissue. Additionally, BSHXF aids in restoring mitochondrial function by modulating antioxidant protein expression, providing a protective effect on NP cells. It helps reduce NP cell apoptosis by down-regulating Bax, cleaved caspase-3, caspase-3, and cytochrome c (cyt-c), while up-regulating B-cell lymphoma 2 (Bcl-2) expression [62]. Both BSHXF and endothelial progenitor cell transplantation significantly contribute to increasing end plate angiogenesis and alleviating IDD by enhancing end plate cells viability, tube-forming capacity, and end plate microcirculation [63].

Research has shown that quercetin alleviates oxidative stress-induced aging in rat NP-derived MSCs via the microRNA-34a-5p (miR-34a-5p)/sirtuin 1 (SIRT1) pathway [64]. It also helps prevent IDD by regulating autophagy through the p38 MAPK pathway [65]. Furthermore, quercetin reduces the expression of senescence-associated secretory phenotype factors and delays senescence in NPCs, thereby mitigating IDD progression through the Nrf2/NF-κB axis [15]. Genistein, a natural isoflavone abundant in soybeans and legumes, has multiple biological activities, including anti-inflammatory, antioxidant, anti-cancer, and bone/cartilage protective effects. It may protect against IDD by inhibiting p38 phosphorylation, thereby reducing inflammation via the p38 pathway [16]. Studies on a rat IDD model have also shown that Genistein can effectively slow IVD tissue degeneration [66]. An in vitro and in vivo study found that genistein protects IDD from degeneration through an Nrf2-mediated antioxidant defence system [67]. Apigenin, a natural small-molecule compound, has demonstrated protective effects against IDD both in vitro and in vivo. It inhibits oxidative stress-induced apoptosis and senescence in NP cells, prevents ECM degradation, and restores autophagic flux disrupted by oxidative stress. This restoration occurs through the promotion of nuclear translocation of transcription factor EB (TFEB) via the AMPK/mTOR signalling pathway, which enhances the function of autophagosomes and lysosomes. In a puncture-induced IDD rat model, apigenin significantly alleviated the progression of IDD, highlighting its potential as an effective therapeutic agent for IDD [17]. Our study found that quercetin simultaneously inhibited CYP1B1 (binding energy −10.0 kcal/mol) and TNFAIP6 (−6.0 kcal/mol) (Figure S7, Panels C and F in the **Online Supplementary Document**), thereby blocking the crosstalk between inflammatory and mechanical signalling. Genistein reduced γδ T cell infiltration (Figure S6, Panel D in the **Online Supplementary Document**), disrupting the ‘target-immune cell’ co-regulatory network. Moreover, the three active compounds present in the ‘Bushen Huoxue Decoction’ exhibited multi-target coverage, mimicking the effects of a natural combination of pathway inhibitors. These findings provide an exploratory hypothesis for future IDD therapeutic strategies; however, further in vitro and in vivo studies are required to clarify their precise mechanisms of action and therapeutic efficacy.

Oral administration of TCM for IDD has demonstrated favourable therapeutic effects. However, the clinical translation of TCM and natural compounds still faces challenges, including low bioavailability, difficulties in standardisation, and a lack of safety evidence. These issues are particularly pronounced in low- and middle-income countries, where implementation of molecular diagnostics and TCM interventions is hindered by underdeveloped health care infrastructure, marked differences in disease spectra, and cultural adaptation barriers. Potential strategies may include tiered diagnosis and treatment systems and the use of locally sourced herbal substitutes. This study provides a theoretical perspective for targeted IDD therapy, but its practical application must follow the principle of ‘contextualised innovation.’ Likewise, the molecular findings of this study are intended to complement rather than replace existing treatment strategies. In aging and resource-limited regions, an integrated approach – combining health education (behavioural modification), ergonomic optimisation (reducing mechanical stress), and policy support (creating an enabling environment) – is essential.

This study aimed to identify potential medicinal herbs at the genetic level and to investigate their molecular mechanisms based on microarray data. Nonetheless, it has certain limitations, including the relatively small sample size of the analysed databases and incomplete database information, which may introduce batch effects and bias in the identification of DEGs. To address these limitations, we plan to expand the data set through additional gene sequencing. It should also be noted that the GO, KEGG, and GeneMANIA databases used in this study carry inherent annotation bias, which may restrict target selection, introduce potential deviations in mechanistic interpretation, and create blind spots in subsequent TCM prediction. In future experiments, we will incorporate single-cell RNA sequencing and spatial transcriptomics to identify region-specific targets and develop an IDD-specific pathway library. Regarding future research directions, given that molecular docking is a predictive method that does not account for the dynamic interactions between proteins and ligands or for in vivo pharmacokinetics, we have already established IDD cell and animal models and initiated relevant pharmacological experiments to validate our findings, with ongoing efforts to elucidate the precise roles of the identified mechanisms.

## CONCLUSIONS

In summary, this study identified CYP1B1 and TNFAIP6 as potential molecular targets for IDD that warrant further validation and developed a nomogram with potential clinical utility, which also requires additional confirmation. Further analysis revealed that these two key targets were involved, to varying degrees, in the regulation of immune infiltration and were closely associated with IDD.

Finally, this study predicted active compounds and TCM formulae corresponding to the key targets and established a ‘gene-compound-formula’ framework for precision treatment, providing preliminary bioinformatics evidence linking specific TCM components to IDD-related molecular targets. These findings offer an exploratory hypothesis and theoretical basis for clinical treatment of IDD, elucidation of its molecular mechanisms, and the efficient development and utilisation of TCM formulae.

## Additional material


Online Supplementary Document
