# Supplementary material for: Utilising bioinformatics and molecular docking technology to explore the underlying mechanisms of intervertebral disc degeneration with potential therapeutic drugs and formulas
Source: J Glob Health. 2025 Nov 21;15:04298. doi: 10.7189/jogh.15.04298 (PMC12635788; doi:10.7189/jogh.15.04298)
Supplement: Online Supplementary Document [file jogh-15-04298-s001.pdf]

Supplement to: Yukui T, Xiaofeng C, Xue B, Lei G, Cheng W, Junchang L. Utilising bioinformatics and molecular docking technology to explore the underlying mechanisms of intervertebral disc degeneration with potential therapeutic drugs and formulas. J Glob Health. 2025;15:04298.

Figure S1. Research flowchart.

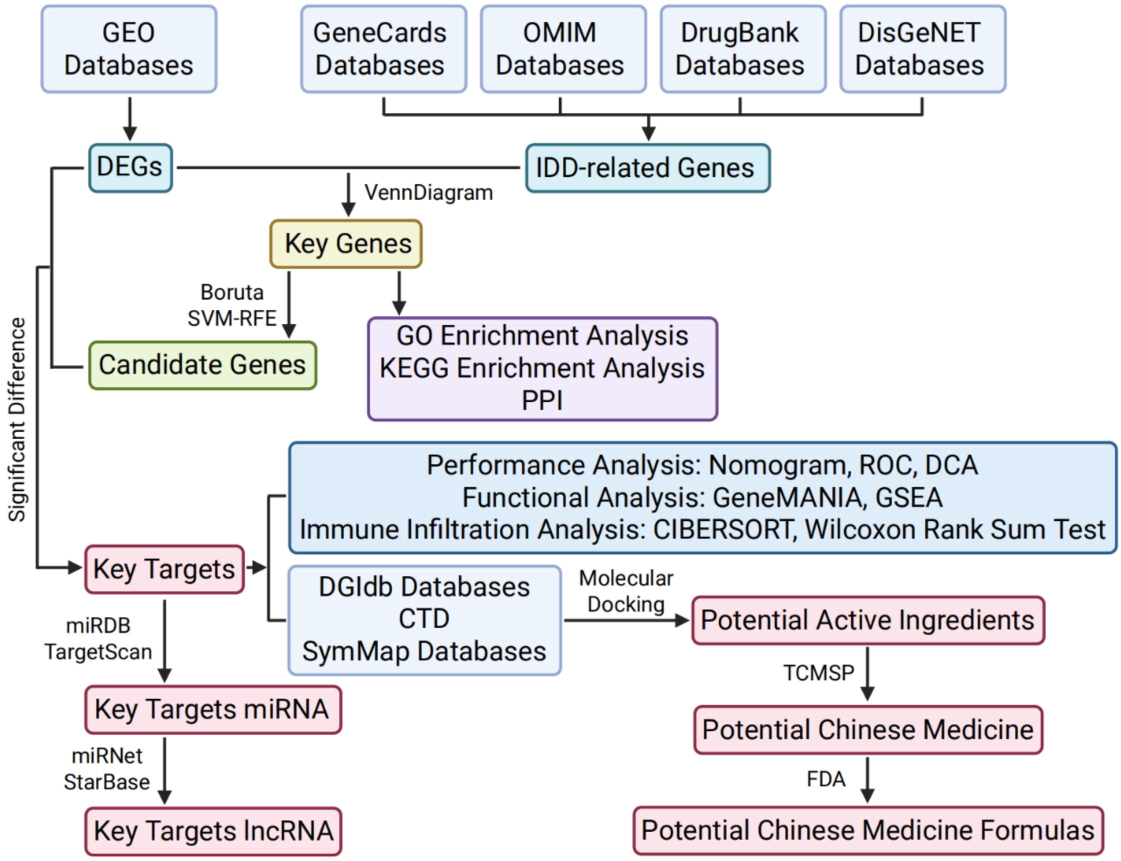

Figure S2. Screening and analysis of IDD candidate genes. **Panel A.** Volcano map of up/ down-regulated circRNAs in IDD. The red dots represent up-regulated circRNAs, and the blue dots represent down-regulated circRNAs. **Panel B.** Heatmap of up/down-regulated circRNAs in IDD. **Panel C.** Venn diagram between IDD-associated disease targets and DEGs. **Panel D.** GO enrichment results. **Panel E.** KEGG enrichment results. **Panel F.** Candidate target PPI map.

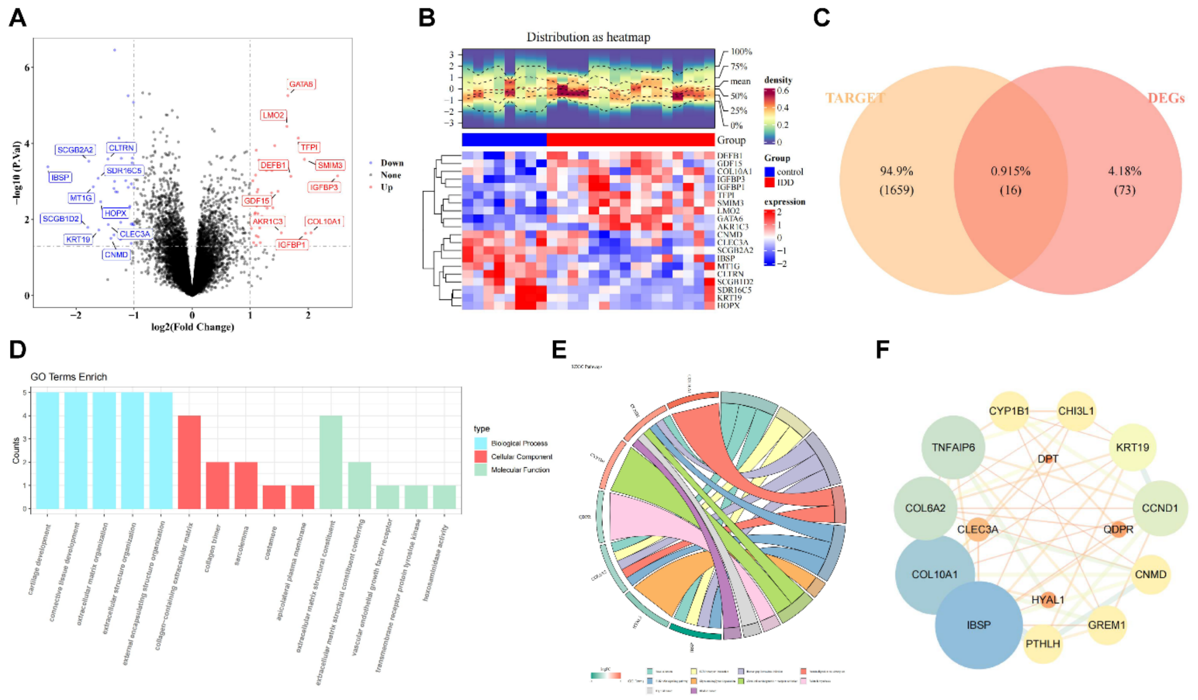

Figure S3. Identification of the key genes. **Panel A.** Boruta algorithm identifies candidate key targets. The horizontal coordinate represents the feature gene, while the vertical coordinate indicates its importance. The green diagram shows Boruta's candidate key targets. **Panel B.** Genetic prediction of true value change curve. The horizontal coordinate is the number of features (number of genes), and the vertical coordinate is the precision of the curve change. **Panel C.** Venn diagram between Boruta feature genes and SVM-RFE feature genes. **Panel D.** Boxplot of expression of candidate key targets in the training set, and expression levels of 7 candidate key targets (CCND1, CYP1B1, HYAL1, COL6A2, TNFAIP6, IBSP, PTHLH) in IDD and normal samples ($p < 0.05$). **Panel E.** Boxplot of the expression of candidate key targets in the validation set, and the expression levels of 7 candidate key targets (CCND1, CYP1B1, HYAL1, COL6A2, TNFAIP6, IBSP, PTHLH) in IDD and normal samples ($p < 0.05$).

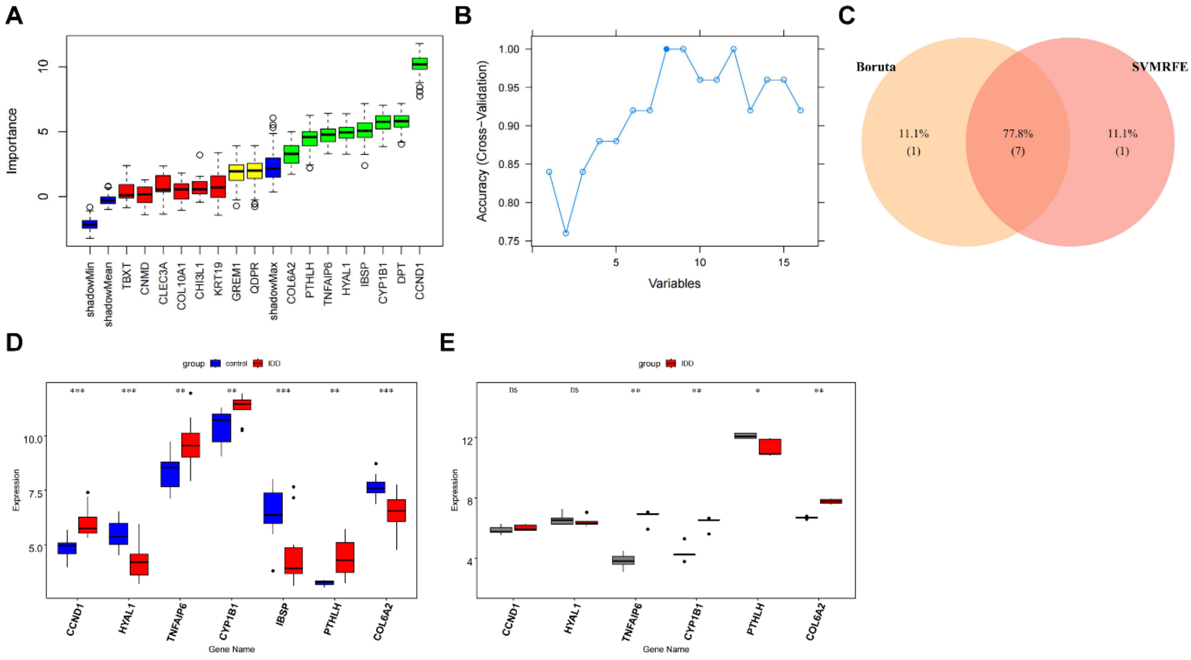

Figure S4. Prediction and diagnosis of IDD by key genes. **Panel A.** ROC curve of CYP1B1 in the training set with an AUC value of 0.906. **Panel B.** ROC curve of TNFAIP6 in the training set with an AUC value of 0.875. **Panel C.** ROC curve of CYP1B1 control in the validation set, AUC value of 1. **Panel D.** ROC curve of TNFAIP6 control in the validation set, AUC value of 1. **Panel E.** The key target was used to construct the nomogram model, and the length of the line segment reflected the contribution of the factor to the outcome event. The single-item score Points in the figure represent the corresponding single-item score of each variable at different values. The Total Points in the figure represent the total score of the corresponding single-item scores after the values of all variables are added up. In the figure, the Risk of Case represents the risk of sample disease (IDD). **Panel F.** Nomogram calibration curve: the abscissa represents the probability of HFpEF predicted by the nomogram, and the ordinate represents the actual probability of HFpef. Perfect predictions correspond to black dashed lines. The solid blue line represents the actual prediction results, and the solid black line represents the prediction results after bias correction by Bootstrapping (1000 replicates). **Panel G.** ROC curve of the nomogram model, AUC value of 0.922. **Panel H.** DCA decision curve. None indicates that all samples are negative (Pi < Pt) with zero net benefit. ALL indicates that all samples are positive, and the net benefit is a backslash with a negative slope.

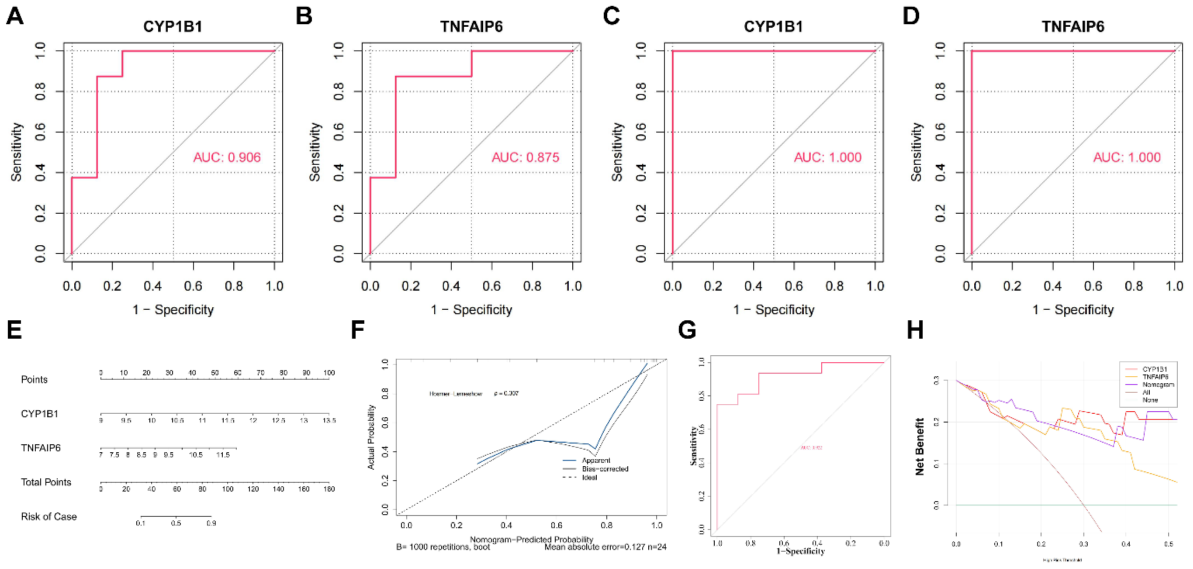

Figure S5. Prediction and diagnosis of IDD by key genes. **Panel A.** GeneMANIA database analysis network diagram, the middle large circle is the key target, and the outer small circle is the genes related to the key target. The right network lines represent, from top to bottom, physical interaction, co-expression, prediction, co-localization, genetic interaction, pathway, and shared protein domains. **Panel B.** KEGG enriched signalling pathways for CYP1B1, top: The top five broken lines are line plots of gene Enrichment scores. The vertical axis represents the corresponding Running ES. The peak in the figure corresponds to the enrichment score of the gene set, and the gene preceding it is the core gene under the gene set. The horizontal axis represents each gene in this gene set. Middle: Hits, where each vertical bar pair is a gene under the gene set. Bottom: Sequencing of genes. **Panel C.** KEGG enriched signalling pathways for TNFAIP6, top: The top five broken lines are line plots of gene Enrichment scores. The vertical axis represents the corresponding Running ES. The peak in the figure corresponds to the enrichment score of the gene set, and the gene preceding it is the core gene under the gene set. The horizontal axis represents each gene in this gene set. Middle: Hits, where each vertical bar pair is a gene under the gene set. Bottom: Sequencing of genes.

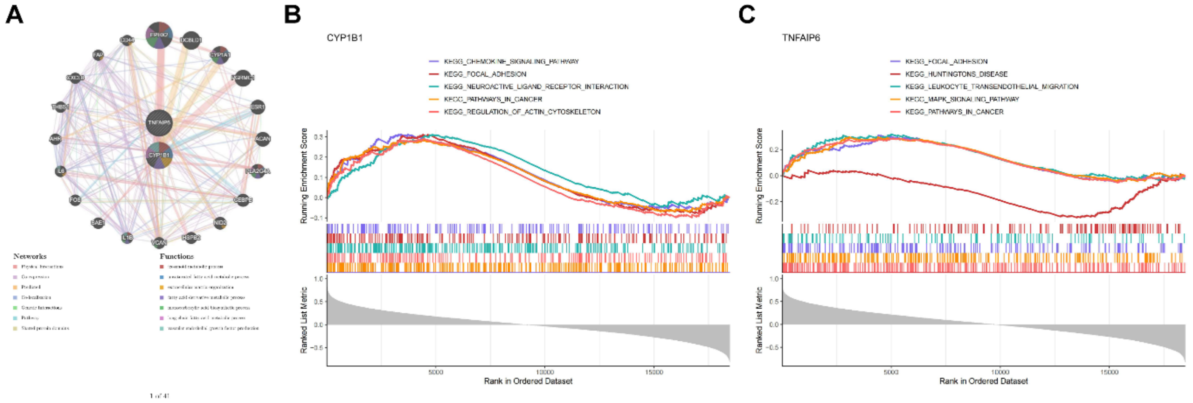

Figure S6. Analysis of the immune infiltration between the IDD and the Control. **Panel A.** Immune cell infiltration abundance plot, evaluating the content of 22 immune cells (infiltration abundance) in IDD samples and normal control samples in the training set. Different colors represent different immune cells. **Panel B.** Significantly different immune cell map, T cell CD4+ memory resting, and T cell gamma delta have significant differences between groups ($p < 0.05$). **Panel C.** Lollipop plot of correlation between CYP1B1 and immune cells, the key target CYP1B1 was significantly correlated with immune cells T cell gamma delta, Macrophage M0, Eosinophil, and B cell memory. **Panel D.** Lollipop plot of the correlation between TNFAIP6 and immune cells, TNFAIP6 was significantly correlated with T cell gamma delta, Macrophage M0, and B cell memory.

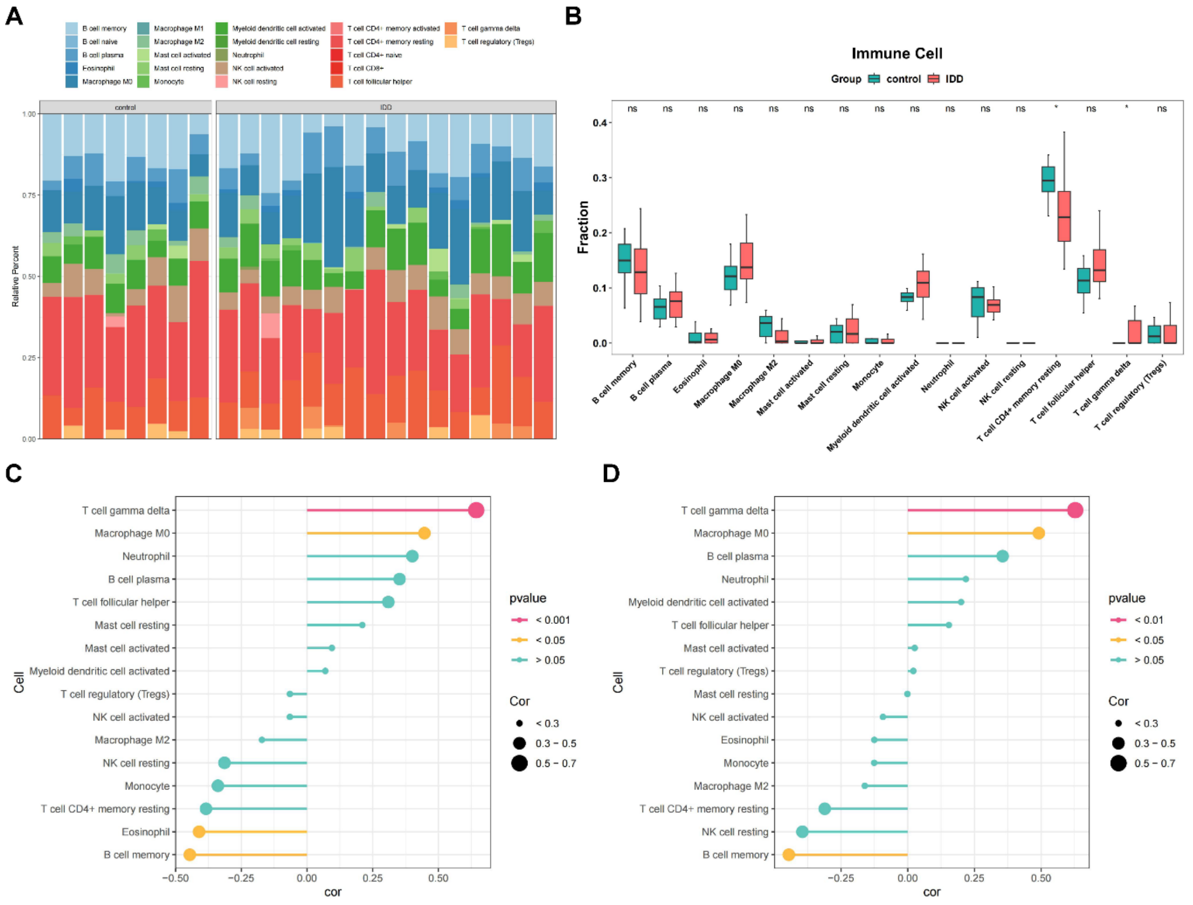

Figure S7. Construction of regulatory networks for key genes and prediction of potential drugs. **Panel A.** ceRNA regulatory network, yellow for key targets, blue for miRNAs, and orange for lncRNAs. **Panel B.** "Potential compound - potential Chinese medicine - meridian - candidate prescription" network. **Panel C.** Molecular docking map of CYP1B1 and Genistein, left for overall molecular docking and right for local magnification of docking. In the figure, the yellow dashed line represents the hydrogen bond force, the blue line denotes the small molecule compound, the dark blue line indicates the protein structure, and the black text highlights the amino acid residues and positions of the small molecule compound and the protein connected by hydrogen bonds. **Panel D.** Molecular docking map of CYP1B1 and Quercetin, left for overall molecular docking and right for local magnification of docking. In the figure, the yellow dashed line represents the hydrogen bond force, the blue line denotes the small molecule compound, the dark blue line indicates the protein structure, and the black text highlights the amino acid residues and positions of the small molecule compound and the protein connected by hydrogen bonds. **Panel E.** Molecular docking map of CYP1B1 and Apigenin, left for overall molecular docking and right for local magnification of docking. In the figure, the yellow dashed line represents the hydrogen bond force, the blue line denotes the small molecule compound, the dark blue line indicates the protein structure, and the black text highlights the amino acid residues and positions of the small molecule compound and the protein connected by hydrogen bonds. **Panel F.** Molecular docking maps of TNFAIP6 and Genistein, with overall molecular docking on the left and local magnification of the docking on the right. In the figure, the yellow dashed line represents the hydrogen bond force, the blue line denotes the small molecule compound, the dark blue line indicates the protein structure, and the black text highlights the amino acid residues and positions of the small molecule compound and the protein connected by hydrogen bonds.

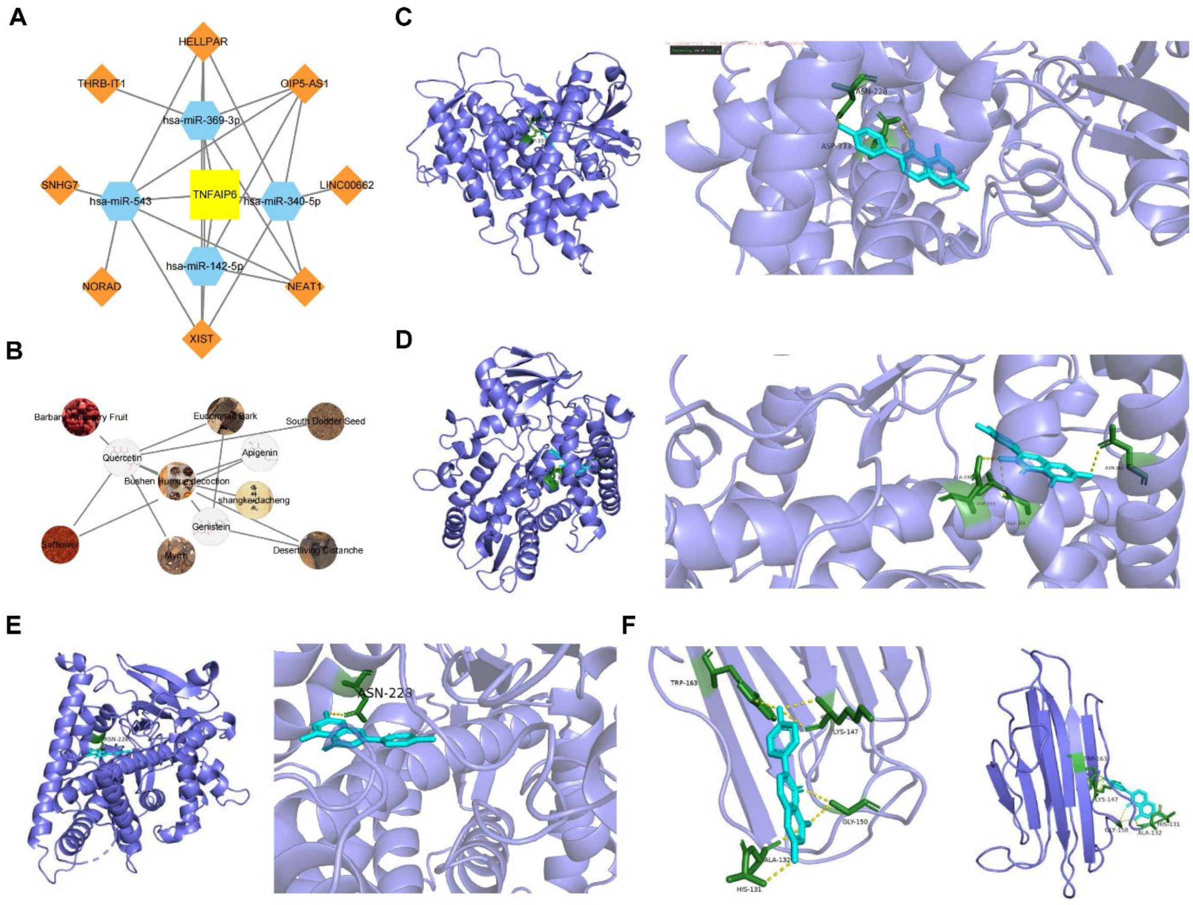
A
B
C
D
E
F
HELLPAR
THRB-IT1
OIP5-AS1
hsa-miR-369-3p
SNHG7
LINC00662
TNFAIP6
hsa-miR-543
hsa-miR-340-5p
hsa-miR-142-5p
NORAD
NEAT1
XIST
Quercetin
South Dodder Seed
Apigenin
shangkedacheng
Genistein
Desertliving Cistanche
ASN-228
ASN-223
TRP-163
LYS-147
GLY-150
ALA-132
HIS-131
